# Phytomediated Biostimulation of the Autochthonous Bacterial Community for the Acceleration of the Depletion of Polycyclic Aromatic Hydrocarbons in Contaminated Sediments

**DOI:** 10.1155/2014/891630

**Published:** 2014-08-07

**Authors:** Simona Di Gregorio, Alessandro Gentini, Giovanna Siracusa, Simone Becarelli, Hassan Azaizeh, Roberto Lorenzi

**Affiliations:** ^1^Department of Biology, University of Pisa, 56126 Pisa, Italy; ^2^Teseco SpA, Via Carlo Ludovico Ragghianti 12, 56121 Pisa, Italy; ^3^STA srl, Via della Gherardesca 2, Ospedaletto, 56121 Pisa, Italy; ^4^Institute of Applied Research (Affiliated with University of Haifa), The Galilee Society, P.O. Box 437, 20200 Shefa-Amr, Israel; ^5^Tel Hai College, 12208 Upper Galilee, Israel

## Abstract

Polycyclic aromatic hydrocarbons (PAHs) are a large group of organic contaminants causing hazards to organisms including humans. The objective of the study was to validate the vegetation of dredged sediments with *Phragmites australis* as an exploitable biostimulation approach to accelerate the depletion of PAHs in nitrogen spiked sediments. Vegetation with* Phragmites australis* resulted in being an efficient biostimulation approach for the depletion of an aged PAHs contamination (229.67 ± 15.56 *μ*g PAHs/g dry weight of sediment) in dredged sediments. *Phragmites australis* accelerated the oxidation of the PAHs by rhizodegradation. The phytobased approach resulted in 58.47% of PAHs depletion. The effects of the treatment have been analyzed in terms of both contaminant depletion and changes in relative abundance of the metabolically active Gram positive and Gram negative PAHs degraders. The metabolically active degraders were quantified both in the sediments and in the root endospheric microbial community. Quantitative real-time PCR reactions have been performed on the retrotranscribed transcripts encoding the Gram positive and Gram negative large *α* subunit (RHD*α*) of the aromatic ring hydroxylating dioxygenases. The Gram positive degraders resulted in being selectively favored by vegetation with *Phragmites australis* and mandatory for the depletion of the six ring condensed indeno[1,2,3-cd]pyrene and benzo[g,h,i]perylene.

## 1. Introduction

Polycyclic aromatic hydrocarbons (PAHs) are a large group of organic contaminants deriving principally from anthropogenic sources such as the uncompleted combustion of fossil fuels, natural combustions, and volcanic eruptions, but with the majority due to anthropogenic emissions such as automobile exhausts, processing production, and accidental spillage of petroleum [1–3]. The United State Environmental Protection Agency (US-EPA) and the European Environmental Agency (EEA) listed 16 PAHs as priority pollutants for their toxicity, mutagenicity, carcinogenicity, and environmental persistence. Due to their low water solubility, high hydrophobicity, complex chemical structure, and recalcitrance to biodegradation, PAHs tend to accumulate in the soil and sediment organic matter. High concentrations of PAHs in soils and sediment cause significant hazards to many organisms comprising humans [4, 5]. Despite their low bioavailability, biodegradation is the principal process affecting the fate of PAHs in the environment. The first hydroxylation step of the PAH ring is crucial to prime the oxidation and eventually the depletion of the contaminants in the different matrices [6]. The reaction consists of incorporation of molecular oxygen into the PAH aromatic nucleus by a multicomponent aromatic ring hydroxylating dioxygenase (RHD) enzyme. The enzyme is composed of large *α* and small *β* subunits [7]. The large *α* subunit (RHD*α*) contains two conserved regions: the [Fe2-S2] Rieske centre and the mononuclear iron-containing catalytic domain. Homologous *α* subunits, encoded by phylogenetically distant genes, have been cloned in both Gram positive (GP) and Gram negative (GN) bacterial species [8]. The presence of these genes is generally considered as representing the PAH-biodegradation potential of environmental matrices, and, in soils and sediments, their quantification is proportional to the level of PAH contamination [6]. In relation to sediments, the depletion of PAH contamination is very challenging. In fact, every year, authorities dispose several hundred million tons of sediments, dredged from the bottom of coastal and internal waterways and harbors. Large portions of these dredged sediments are contaminated by organic compounds, comprising PAHs. Due to the huge amount of sediments to be treated, the confined disposal in dedicated area and the monitored natural attenuation of the contamination are currently the more common and actually affordable strategy adopted to treat them. The monitored natural attenuation can be associated to significant risks for the community because of possible leakages and the consequent deterioration of the surrounding environment. Thus, the acceleration of the process and the fast safe reallocation of decontaminated sediments are a priority for the protection of the environment and the community. Biostimulation consists mainly of accelerating the processes of natural attenuation of the contamination by favoring the metabolic activity of the autochthonous microbial community, eventually competent for contaminant transformation. Among biostimulation approaches, vegetation of different contaminated matrices with plant species found applications for accelerating the depletion of disparate types of very recalcitrant contaminants comprising PAHs [9, 10]. The process is defined as rhizoremediation and it is mainly due to the stimulation of the biodegradation of the organic contaminants by rhizobacteria [11–15] through root exudates [16, 17] that can eventually increase the bioavailability of the contaminants and induce a natural selection of the bacterial rhizospheric communities [18–20]. Moreover, in relation to saturated dredged sediments, plants, by exploring the matrix with oxygen-transporting roots, offer the further advantage of exposing the anoxic sediments to aerobic conditions, eventually favoring the oxidation of the contaminants. However, the efficiency of a phytoremediation process depends mainly on the presence and activity of plant-associated microorganisms carrying degradation genes required for the enzymatic breakdown of contaminants. These microorganisms have been recorded, both in the plant rhizosphere and in the plant endosphere [20–22]. The composition of the rhizospheric (microbial communities colonizing the surrounding of the roots) and the endospheric microflora (microbial communities colonizing plant tissues) is known to be dependent on both plant and soil type [23–26]. In case the plant is growing in presence of contaminants, there is an interaction between contaminant, soil, and plant species that determines the structure and the metabolic activity of the plant associated microbial communities [20, 27, 28]. The knowledge of this interaction is far from being complete, even though it is essential for designing an efficient plant-based biostimulation of contaminants biodegradation.

The objective of the current study consisted of validating the vegetation of dredged sediments with the highly transpiring plant* Phragmites australis* [29] as an exploitable biostimulation approach to accelerate the depletion of PAHs. In addition, the quantification of the levels of transcription of the bacterial Gram positive and Gram negative PAH-RHD*α* genes encoding the enzymes priming the PAH oxidation has been performed, both in bulk and rhizospheric portion of the sediments and in the root endophytic microbial community. This is the first report on the capacity of* P. australis* to promote the metabolic activity of both rhizospheric and endophytic Gram positive degraders on a vast array of PAHs eventually comprising the high molecular weight compounds.

## 2. Material and Methods

### 2.1. Chemicals, Plants, and Sediments

Chemicals used throughout the experiments were of analytical grade and purchased from Sigma-Aldrich (Milan, Italy).* Phragmites australis* (reed) plants were collected from a local nursery at the same developmental stage. Chemical standards of the 16 PAHs including naphthalene (NA), acenaphthylene (ACY), acenaphthene (ACE), fluorene (FL), phenanthrene (PH), anthracene (AN), fluoranthene (FLU), pyrene (PY), benzo[a]anthracene (BaA), chrysene (CH), benzo[b]fluoranthene (BbF), benzo[k]fluoranthene (BkF), benzo[a]pyrene (BaP), indeno[1,2,3-cd]pyrene (IP), dibenzo[a,h]anthracene (DA), and benzo[g,h,i]perylene (BP), the deuterated PAH internal standard solutions (naphthalene-d8, acenaphthene-d10, phenanthrene-d10, chrysene-d12, and perylene-d12), and surrogate standard solutions (2-fluorobiphenyl and 4-terphenyl-d14) were obtained from AccuStandard Chem. Co. (USA). Internal and surrogate standards were used for sample quantification and process recovery.

PAH contaminated sediments (229.67 ± 15.56 *μ*g PAH/g dry weight of sediment) (Table 1) have been collected from a coastal artificial aquifer at the estuary of a river crossing an industrialized area in the North of Italy (44°29′22′′N; 12°15′52′′E). The texture of the sediments was sandy loam (35% silt, 50% sand, and 15% clay) with total phosphorous of 1.5% and total nitrogen of 1.3%.

### 2.2. Experimental Conditions

A total of 42 experimental replicates, each containing 25 kg of air-dried dredged sediments, were prepared in stainless steel pots. The pots were maintained at 24 ± 1°C under controlled growth chamber (14 h light/10 h dark). Sediments in each pot were saturated up to the 80% of the maximum water-holding capacity. The water content was maintained by monitoring the constant weight for each replicate, eventually adding tap water anytime evapotranspiration decreased the percentage of sediment retained water. Evapotranspiration was the only possibility of water loss for the experimental pots. A total of 36 pots out of the 42 were spiked with NH_4_NO_3_ (N spiked) which was dissolved in water to maintain a C : N ratio of 10 : 1. The other 6 pots, not N spiked (not biostimulated), were analyzed after 12 months of incubation in the growth chamber to represent the controls of the biostimulation process. A total of 18 N spiked pots were vegetated, each with a total of 5* P. australis* plants per pot (average plant age 1 year). After 6, 9, and 12 months of incubation, six vegetated pots for each time-point were processed for chemical analysis. In parallel, after 6, 9, and 12 months, a total of six N spiked, not vegetated pots, for each time-point were also processed. The not vegetated pots were roughly mixed every month before being processed. The redox potential of sediments was monitored every 3 days using a redox tester (HI 98120, HANNA instruments, Italy), recording the parameter in different points per pots up to the stabilization of the recorded values.

### 2.3. PAH Extraction and Analytical Procedures

Sediments and plant samples were collected and analyzed for PAH content, derived from the independently destructive dissection of 6 pots per time-point of analysis. From each pot, plants were pulled out and representative subsamples were prepared, consisting of roots and shoots portions. The total dry mass of the plant was determined by applying the dry/wet factor from the subsampled tissues to the total wet biomass. The sediments in each pot were roughly mixed and a total of 10 different samples of 0.5 Kg of sediments were randomly collected. The different 10 sediment samples per pot were mixed together and the resulting 5 Kg of sediments was divided into two parts and separately extracted and analyzed. The two parts were separately analyzed as replicates. Sediment and plant samples were dried in a vented oven at 25°C for 36 hrs. All samples were extracted using a soxhlet apparatus with 1 : 1 (v/v) acetone/*n*-hexane. A total of 10 *μ*L of surrogate standard mixture (2-fluorobiphenyl and 4-terphenyl-d_14_) solutions was added. The concentration of the surrogates was 250 ng/mL each. Method blanks were prepared following the same procedure without adding sediment sample. The verification of the calibration for quality control was prepared by adding the standard solution to 1 : 1 (v/v) acetone/*n*-hexane at a concentration equal to the 75% of the last calibration point. After subsequent drying over anhydrous sodium sulphate of the organic fraction and concentration to 1.0 mL using a gentle stream of nitrogen, an internal standard mixture (naphthalene-d_8_, acenaphthene-d_10_, phenanthrene-d_10_, chrysene-d_12_, and perylene-d_12_) solution at a final concentration of 50 ng/mL each was added to the extract to be analyzed using gas chromatography with mass selective detection (GC-MS). The internal standard mixture has been selected to cover all the fragmentation range of the 16 analyzed PAHs. The quantitative analysis of PAH was performed with an Agilent 6890 GC-5975B Series MS system in the selective ion monitoring mode (SIM). Injection of 1 *μ*L of samples was conducted in the splitless mode with a sampling time of 1.0 min. Separation of PAH congeners was carried out with a 30 m (long) × (0.25) mm I.D. (inner diameter) HP-5MS capillary column (Hewlett-Packard, Palo Alto, CA, USA) coated with 5% phenyl-methylsiloxane (film thickness 0.25 *μ*m). The injection temperature was 300°C. The transfer line and ion source temperatures were 280°C and 200°C, respectively. The column temperature was initially held at 40°C for 1 min and raised to 120°C at the rate of 25°C/min, then to 160°C at the rate of 10°C/min, and finally to 300°C at the rate of 5°C/min, held at final temperature for 15 min. Detector temperature was kept at 280°C. Helium was used as a carrier gas at a constant flow rate of 1 mL/min. Identity of the PAHs in the samples was confirmed by retention time and the relative abundance of selected monitoring ions of the standard PAHs. The 16 priority PAHs were quantified using the response factors related to the respective internal standards based on five-point calibration curve for each individual compound ranging from 10 ng/mL to 1000 ng/mL. Each PAH concentration was corrected using the recovery rate of the surrogate standard and expressed on a dry-weight basis.

### 2.4. Bacterial Community Structure

The rhizospheric portion of the sediments as well as the portion of sediment adhering to the root apparatus of the 5 plants that vegetated in each pot was collected. A total of 30 different sediment samples per plant were collected. Each sample accounted for a total of 250 mg. All samples collected from each pot were thoroughly mixed before sampling the 250 mg of sediment to be processed for the extraction of the rhizospheric total community RNA. The bulk portion of sediments in the vegetated pots as well as in the not vegetated pots was collected by thoroughly mixing the sediments in each pot after intact plants have been pulled out from the pots. A total of 50 different samples of 250 mg of sediments per pot were randomly collected and mixed before sampling the 250 mg of sediments to be processed for the extraction of the bulk total community RNA. The total RNA from sediments was purified using the MoBio RNA power soil total RNA isolation kit (MoBio Laboratories Inc., USA) following the manufacturer's instructions. Total RNA from plant roots was isolated using RNEASY Plant Mini Kit (Qiagen, Italia). Tissues were sampled before the plants were planted in the pots and at each time-point of analysis. A total of 3 different root samples per plant were collected. Each sample accounted for a total of 2.5 g. The samples were finely chopped, transferred to a 500-mL Erlenmeyer flask containing 200 mL of NaClO (1.05% v/v) in sterile water, and placed on a rotary shaker (200 rpm) at 22°C for 10 min. Roots were rinsed 4 times with 200 mL of sterile PBS, and 0.1 mL of the final wash was diluted in 9.9 mL of 1/10 TSB to test for rhizospheric contamination [31]. The roots were then chopped into less than 1 mm sections and roughly mixed and a total of 1 mg of root tissues was processed. Potential DNA contamination in the total RNA from sediments was eliminated with DNase I treatment using the RiboPure bacteria kit (Ambion, USA) according to the instructions of the manufacturer. The potential presence of contaminating DNA was tested by PCR amplification of 16S rDNA. RNA quantity, quality, and purity were analyzed using gel electrophoresis on a 1% (w/v) agarose gel stained with ethidium bromide and viewed spectrophotometrically with an Implen nanophotometer (Implen GmbH, Germany). To produce cDNA template for the PCR amplification of the PAH-ring hydroxylating dioxygenases retrotranscript and rcRNA, reverse transcription was performed on the total community RNA extracted, using the RNase H activity-less RevertAid premium reverse transcriptase (Fermentas, Lithuania) according to the instructions of the manufacturer. The absence of contaminating DNA was confirmed by using nontranscribed RNA as template in PCR amplification. Primers used for reverse transcription and the quantification of the transcript for PAH-ring hydroxylating dioxygenases and total 16S rDNA are listed in Table 2.

Quantitative real-time PCR reactions were carried out on an ABI Prism 7300 Sequence Detection System using Sybr Green PCR Master Mix (Applied Biosystem, Monza, Milan, Italy). The presence of PCR inhibitors was estimated by diluting the cDNA and by mixing a known amount of standard DNA to the cDNA to detect any PCR reaction failure indicating the presence of PCR inhibitors. No inhibition was detected in both cases. For each rtPCR reaction, approximately 2 ng of cDNA was used as template; forward and reverse primers were used at a 0.6 mM final concentration and water to a final volume of 50 *μ*L. Samples were always measured in triplicate. Each 25 *μ*L reaction contained 0.3 *μ*mol of each primer, 12.5 *μ*L Maxima SYBR Green qPCR Master Mix (Fermentas, Vilnius, Lithuania), and 5.0–10.0 ng cDNA (Table 2). The cDNA samples were diluted 1 : 10 to reduce the effect of possible PCR inhibition. Thermal cycling conditions were 10 min at 95°C, 42 cycles of 15 s at 95°C, 30 s at the specific annealing temperature (Table 2), and 30 s of elongation at 72°C, and during real-time PCR alone the SYBR Green I signal intensities were measured during a 10 s step at 80°C to dissociate the primer's dimers. The final step consisted of 7 min at 72°C. A melting curve ranging from 50 to 99°C was performed with steps of 1°C and a hold of 5 s to check the specificity of the assays. For each amplified target, a standard curve was established using serial dilutions of linearized plasmid pGEM-T (10^2^ to 10^7^ copies) containing cloned PAH-ring hydroxylating dioxygenase genes and 16S rDNA. The linearized plasmid for GN PAH-RHD*α* quantification was constructed by cloning the corresponding purified PCR product (QIAquick PCR purification kit, QIAGEN) from* Pseudomonas putida* G7 DSM 50222. For the GP PAH-RHD*α* and 16S rDNA quantification, the linearized plasmids were constructed by cloning the corresponding PCR products from* Mycobacterium vanbaalenii* DSM 7251. Two nontemplate controls (NTCs) were also included in all the assays. At each run, two standard curves per sample were included and all the samples were related to the corresponding standard curve. Amplification efficiencies were calculated from the slopes of the standard curves. To confirm the specificity of each of the primer sets, samples from the first run using the primer set were loaded onto a 2% agarose gel. Primer sets did not yield any side products in low copy number dilutions and in NTCs. The amplification levels of the retrotranscript of the PAH-ring hydroxylating dioxygenase genes were first normalized by the amplification level of 16S rcDNA. The fractional copy number of each amplified gene was calculated as the ratio between the normalized amplification levels for each gene in (i) N spiked sediments, (ii) N spiked and vegetated sediments, and the normalized amplification levels of the genes in not biostimulated (N spiked and not vegetated) sediments. All the results were analyzed with ABI Prism 7300 software v1.3.1.

### 2.5. Statistical Analysis

All the data were analyzed with the aid of ANOVA, and the means were separated using the Bonferroni correction test (*P* ≤ 0.05) and by applying the specific software Statgraphics 5.1 (Statistical Graphics Corp., USA).

## 3. Results

### 3.1. PAHs Depletion

**Figure 1:**
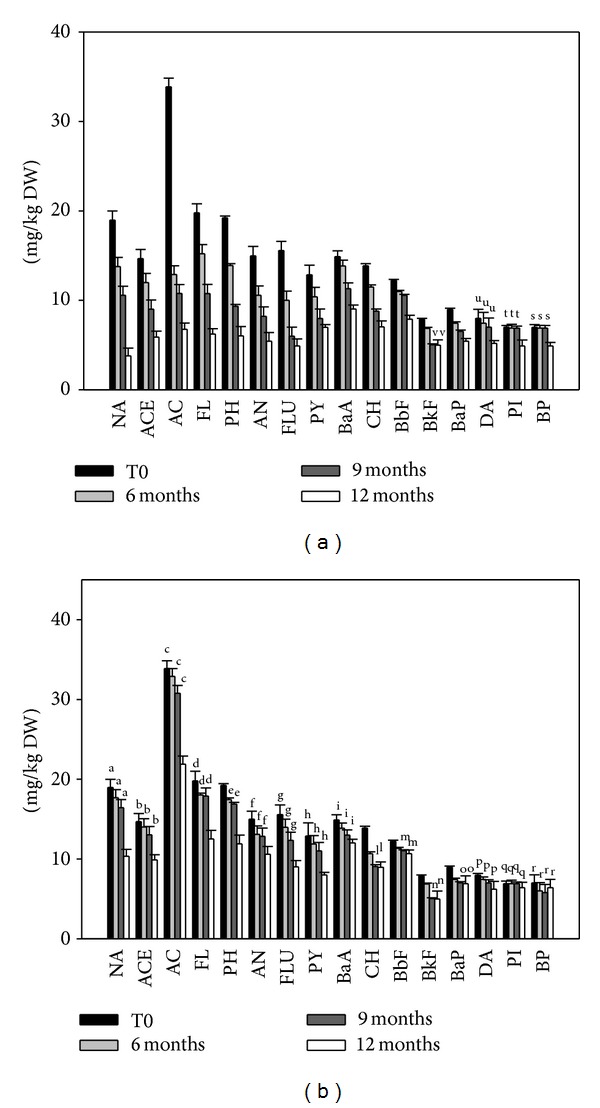
The concentrations of the different PAHs (see abbreviations in Material and Methods) at the different time of incubation of biostimulated sediments. In (a), the sediments were spiked with N and vegetated with* P. australis* plants. In (b), the sediments were spiked with N only. Histogram values represent mean + SE of the six parallel samples. The same letters on the bars indicate values not significantly different at *P* > 0.05.

As shown in Table 1, after 12 months of incubation, the PAH depletion was absent in the not biostimulated sediments (notB). On the other hand, either in N spiked (Ns) or in N spiked and vegetated sediments (NsV), the PAH depletion was observed (Table 1). In fact, biostimulation induced the progressive depletion of the different PAHs that resulted in being more consistent in the case of vegetation with* P. australis* plant. In N spiked sediments the depletion of the 16 PAHs occurred mainly during the last 3 months of incubation, after a lag phase of 9 months (Figure 1(b)). Moreover, the 6 ring condensed PAHs, the indeno[1,2,3-cd]pyrene (PI), and the benzo[g,h,i]perylene (BP), were not depleted, even after 12 months of incubations. On the other hand, an earlier onset of PAH depletion in vegetated sediment with respect to not vegetated one has been observed (Figure 1(a)). After only 6 months of incubation a significant PAH depletion has been recorded and in vegetated sediments the PAH depletion resulted in being gradual during the 12 months of experimentation with a final depletion of indeno[1,2,3-cd]pyrene (PI) and benzo[g,h,i]perylene (BP) accounting for 29.94% and 29.79%, respectively (Table 1). More in detail (Table 1), after 12 months of incubation, the depletion of naphthalene accounted for the 79.97% in N spiked and vegetated sediment and for the 45.49% in N spiked and not vegetated sediments. With reference to the 3 condensed ring PAHs, the percentage of depletion varied between 59.85 and 80.1% with a mean value of 68.14% in N spiked and vegetated sediments. In N spiked and not vegetated sediments, the percentages of the 3 condensed ring PAHs depletion varied between 29.31 and 38.22% with a mean value of 34.44%. In relation to the 4 condensed ring PAHs, in N spiked and vegetated sediments, the percentage of depletion varied between 39.35% and 68.52% with a mean value of 50.73%. In N spiked and not vegetated sediments, the percentage of depletion accounted for 19.4% and 42.1% with a mean value of 33.67%. The depletion of the 5 condensed ring PAHs in N spiked and vegetated sediments varied between 34.84% and 39.46% with a mean value of 36.65%. In N spiked and not vegetated sediments, the percentages varied between 12.71% and 37.65% with a mean value of 23.57%. In relation to the six condensed ring PAHs in N spiked and vegetated sediments, the percentage of depletion of the PI was 29.94% and the BP was 29.79%. In N spiked and not vegetated sediments, the percentages of depletion of PI and BP were not statistically significant (0.07 < *P* < 0.08) but accounted for 7% for both the PAHs. As a net result, the total PAH depletion (∑  PAHs) accounted for 58.47% in N spiked and vegetated sediments, compared to 31.79% in N spiked and not vegetated sediments (Table 1).

The quantification of the different PAHs in the* P. australis* tissues indicated that, within the limitation of the analytical methods adopted, none of the 16 PAHs has been detected in plant tissues, and the percentages of PAHs removed by plant uptake are negligible. In not vegetated sediments, the redox potential was constantly negative (≈Eh ≤ −217 ± 0.43 mV). In the case of vegetation of sediments with* P. australis*, the related values were between Eh ≤ −202 ± 0.13 mV and Eh ≤ −193 ± 0.52 mV.

### 3.2. Bacterial Community Structure

**Figure 2:**
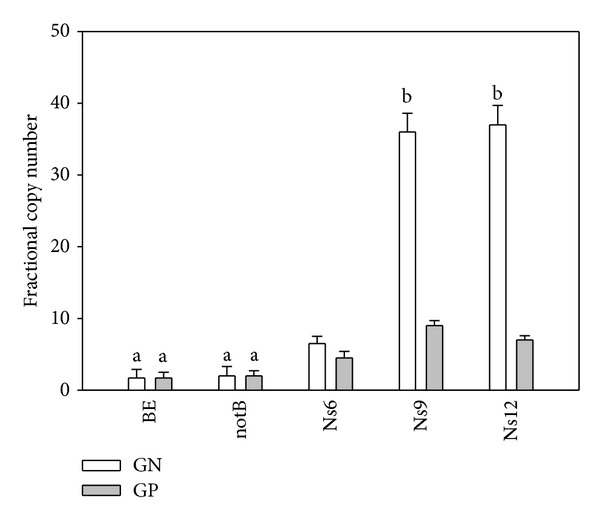
Fractional copy numbers of GN (Gram negative) and GP (Gram positive) PAH-RHD*α* transcripts at the different time-points of analysis in N spiked sediments. Histogram values represent mean + SE of the 6 parallel samples. The same letters on the bars indicate values not significantly different at *P* > 0.05. BE: beginning of the experiment; notB: not biostimulated sediments (not N spiked nor vegetated) after 12 months of incubation; Ns6: N spiked sediments after 6 months of incubation; Ns9: N spiked sediments after 9 months of incubation; Ns12: N spiked sediments after 12 months of incubation.

**Figure 3:**
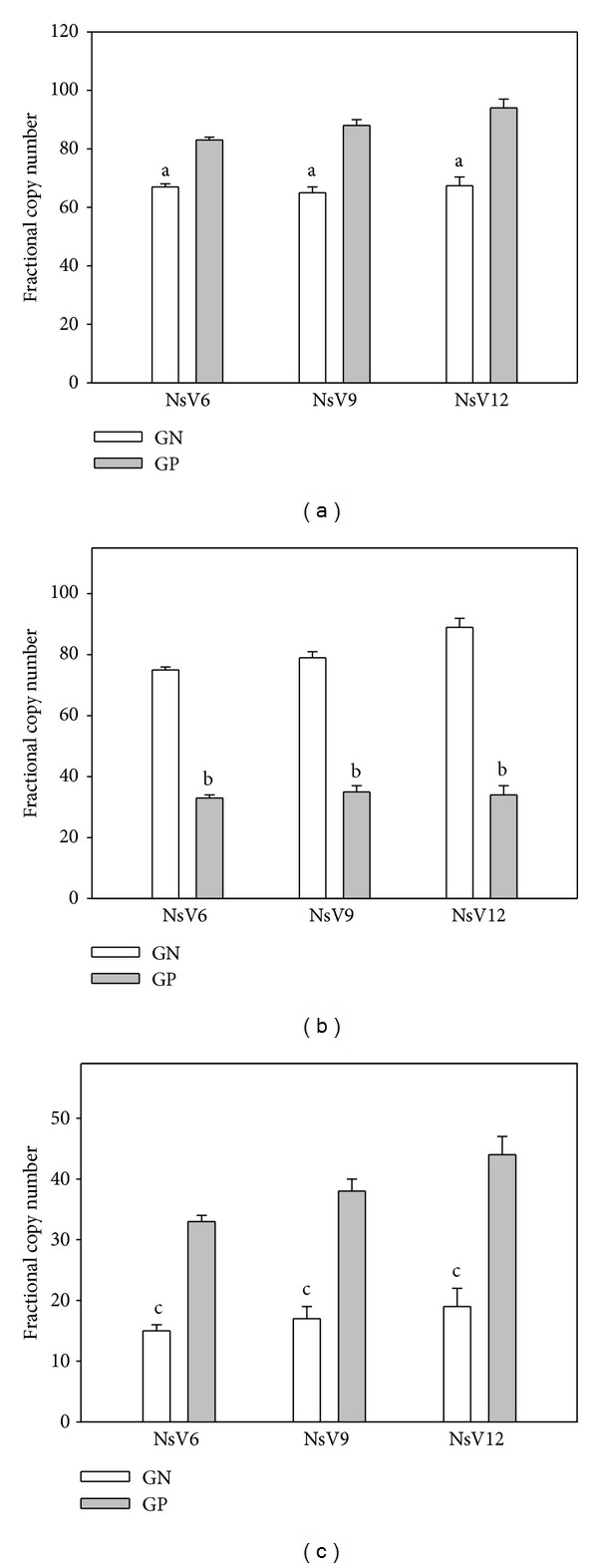
Fractional copy numbers of GN and GP PAH-RHD*α* transcripts at the different time-points of analysis in vegetated sediments. Histogram values represent mean + SE of the 6 parallel samples. The same letters on the bars indicate values not significantly different at *P* > 0.05. NsV6: N spiked and vegetated sediments after 6 months of incubation; NsV9: N spiked and vegetated sediments after 9 months of incubation; NsV12: N spiked and vegetated sediments after 12 months of incubation. (a) Rhizospheric portion of vegetated sediments; (b) bulk portion of vegetated sediments; (c) root endophytic bacterial population of* P. australis* plants.

The quantification of the GP and GN PAH-RHD*α* transcripts was normalized to the levels of transcription of the 16s rDNA representing the portion of the metabolic active bacterial structure. Results obtained are shown in Figures 2 and 3. The GP and GN PAH-RHD*α* transcripts were all detected at the beginning of the experiment (BE) at a level of transcription not significantly different from the one measured in not biostimulated sediments (notB) after 12 months of incubation (0.06 < *P* < 0.07) (Figure 2). On the other hand, the spiking of N into the sediments induced changes in the metabolically active bacterial community structure. With reference to the beginning of the experimentation (BE), after 6 months of incubation, a 3.5 and a 2.4 higher fractional copy number for the GN and the GP PAH-RHD*α* transcripts, respectively, have been detected (*P* < 0.04). The increase has been recorded also after 9 months of incubation when the GN and GP PAH-RHD*α* transcripts were 19.4 and 4.8 higher than the values recorded at the beginning of the experimentation (*P* < 0.05). Compared to 9 months of incubation, after 12 months, no significant increase in the fractional copy number for the GN PAH-RHD*α* transcripts and a slight decrease (22%; *P* = 0.05) in the fractional copy number of the GP PAH-RHD*α* transcript have been recorded (Figure 2).

Results obtained in vegetated sediments are shown in Figure 3. A stronger effect of* P. australis* vegetation on the metabolically active microbial population with reference to the sole N spiking was evident. After 6 months of incubation, the vegetation of N spiked sediments determined an increment, respectively, of 36.2 in rhizospheric (Figure 3(a)) and 40.5 in bulk (Figure 3(b)) portion in the fractional copy number of the GN PAH-RHD*α* transcripts with reference to the beginning of the experimentation (Figure 2). The fractional copy number of the GN PAH-RHD*α* transcripts did not increase with the progression of the incubation period in the rhizospheric portion of the vegetated sediments (0.06 < *P* < 0.07) (Figure 3(a)). On the other hand, an increase in the corresponding fractional copy number has been observed in the bulk portion of the vegetated sediments (*P* < 0.05) (Figure 3(b)), reaching values of 48.1 higher than the one at the beginning of the experimentation. Interestingly, remarkable increase in the fractional copy number of the GP PAH-RHD*α* transcripts has been observed in the rhizospheric portion of the vegetated sediments (*P* < 0.05) (Figure 3(a)). After 6 months of incubation, the corresponding values were 44.8 times higher than the one at the beginning of the experimentation (*P* < 0.05). The increment for the GP PAH-RHD transcript was higher than the one for the GN PAH-RHD transcripts with reference to the beginning of the experimentation (*P* < 0.05). Moreover while these latter did not increase with time of incubation, an increment in the fractional copy number of the GP PAH-RHD transcripts has been observed (0.07 < *P* < 0.08). In relation to the* P. australis* root endosphere (Figure 3(c)), at the beginning of the experimentation, the fractional copy number of the GN and GP PAH-RHD*α* transcripts has been quantified in the plant root used for the vegetation of the sediment. At that time of analysis, any GP and GN PAH-RHD*α* transcript has been recorded. On the other hand, starting from the six months of incubation, a progressive increment in the related fractional copy numbers has been observed, with a net predominance of the GP PAH-RHD*α* transcripts on the GN one.

## 4. Discussion

Dredged sediments from aquifers crossing industrialized areas are generally characterized by the presence of recalcitrant organics, including PAHs that progressively accumulated with time, in years and decades [32, 33]. The PAH contamination recorded here was mainly due to the two, three, and four condensed rings molecular structures. However, very recalcitrant five and six condensed rings PAHs were also present. With the aim to design a bio-based approach for a cost-sustainable depletion of the PAH contamination, a phytobased approach has been adopted. Its efficacy has been evaluated with respect to the sole amendment of a nitrogen source to the sediments, in accordance with the resource-ratio theory, mandatory to favor the metabolic activity of autochthonous microbial communities. The resource-ratio theory consists in one of the major logical frameworks used in ecology to predict how competition for growth-limiting resources influences biological diversity and function within a biological community [34]. More precisely, changes in nitrogen supply ratio conditions can significantly alter biodegradation rates of the organic matter, causing shifts in microbial community composition, comprising the total hydrocarbon degrader biomass. In virtue of the resource-ratio theory and personal observations that, in the case of aged contaminations, the restored nitrogen content is mandatory to biostimulate the natural attenuation of contaminations in soils and sediments, the N spiking into the dredged sediments has been here exploited as the minimal intervention on the sediment autochthonous microbial community, which is necessary to initiate the process of PAH depletion. In fact, results obtained showed that autochthonous microbial populations expressing both the GP and GN PAH-RHD*α* were present in dredged sediments. However, they were probably represented by numerically few candidates that, even though metabolically active, in the frame of the limitation of the analytical method adopted here, did not determine a significant reduction in PAH content in sediments. The N spiking determined an increase in both the GP and the GN metabolic active populations, starting from six months of incubation. The numerical fractional copy number of GN increased significantly after nine months of incubation, reaching a density that positively correlated with the onset of PAH depletion. An increase in the fractional copy number of the metabolically active GP degraders also occurred after six months of incubation, despite reaching lower values with respect to the GN degraders. After nine months of incubation, a slight decrease in the fractional copy number of the GP degraders has been observed. Results obtained suggested that the N amendment restored the nutritional conditions that allowed the autochthonous microbial population, competent for the PAH oxidation, to reach a sufficient density to initiate a process of natural attenuation of the PAH contamination. The mentioned density has been reached after 9 months of incubation and the GN degraders played a pivotal role in the process of PAH depletion. However, any depletion of the six condensed ring PAHs has been recorded in N spiked sediments.

As previously mentioned, in the context of the design of a biobased approach for a cost-sustainable depletion of the PAH contamination in dredged sediments, a phytobased approach has been adopted here to accelerate the process of contaminant depletion. The plant species selected here was the* P. australis*, which, already reported as capable to promote the biodegradation of PAHs in contaminated soils [35], in a reducing environment like dredged sediments, can increase the redox potential of the contaminated matrix, favoring oxidative processes. In fact, considering the high oxygen demand of the PAH catabolism, the delivery of oxygen by plant roots in the anoxic sediments can favor the process of PAH oxidation. The* P. australis* is a high transpiring wetland plant, capable of determining an increase of the redox potential at the root-soil interface because of the transport of oxygen from the aboveground into the underground portion of the plant.* P. australis* plants potentially create the oxidative environments potentially stimulating the aerobic decomposition of the organic matter [36] and, eventually, of the organic contamination in vegetated substrates. In fact, in this experimentation, an increase of the redox potential in vegetated sediments has been observed with reference to not vegetated one. The increase in the redox potential positively correlated with the onset of the PAH depletion that, in the case of vegetated sediments, started after only six months of incubation. As a matter of fact, vegetation of the N spiked sediments resulted in the acceleration of the process of PAH depletion. Similar effects have been observed on pyrene depletion in sediments vegetated with* P. australis* that was also accompanied with a plant-induced increase in the number of pyrene-degrading bacteria [37]. Actually, also in the present experimentation, an increment in the fractional copy number of the GP and GN PAH-RHD*α* transcripts in vegetated sediments has been observed. The highest values in copy numbers of the PAH-RHD*α* transcripts have been observed in response to vegetation of sediments with* P. australis*. More interestingly, the vegetation of the N spiked sediments induced also the depletion of the six condensed rings PAHs. In fact, the vegetation of sediments determined a general increase in the fractional copy numbers of the PAH-RHD*α* transcripts with respect to not vegetated sediments, indicating a general higher bacterial metabolic activity. In the bulk portion of vegetated sediments the metabolically active GN degraders were numerically more represented than the GP one, as observed in N spiked sediments. On the other hand, in the rhizospheric portion of vegetated sediments, the metabolically active GP degraders were numerically more represented than the GN one. The net increase in GP metabolically active degraders, observed only in vegetated sediments, positively correlated with the depletion of the six condensed ring PAHs, the indeno[1,2,3-cd]pyrene, and the benzo[g,h,i]perylene, suggesting the involvement of those bacterial candidates in the depletion of the high molecular weight PAHs. The GP PAH degraders are reported as dominating in aged PAH-polluted sites [38], where bioavailability of PAHs is very low. In fact, the majority of initial PAH degradation could be done by r-strategists GN degraders [39], whereas k-strategists GP bacteria could compete for the biodegradation of more persistent PAHs because of their capacity to increase their bioavailability [40, 41]. Actually, it is reasonable to assume that the sediments analyzed here were facing a case of aged PAH contamination accompanied by the presence of both metabolically dormant GN r-strategists and k-strategists GP bacteria. The increase in availability of PAHs due to the solubilizing activity eventually exerted by* P. australis* could have favored the GP k-strategists with reference to the GN r-strategists. In fact, a positive chemotactic interaction between* P. australis* and the GP degraders cannot be excluded, because of the observed onset of a metabolically active population of GP degraders in the* P. australis* root endospheric microbial community, significantly exceeding the GN one. Interestingly, the metabolically active GP fraction of PAH degraders reached higher values with reference to the GN fraction, not only in the rhizospheric portion of vegetated sediments, but also in the root endosphere of* P. australis* root. These increases in GP degraders were mandatory for the six condensed ring PAH depletion. As a matter of fact,* P. australis* determined an increase in GP degraders in N spiked sediments.

On the other hand, in the frame of the limitation of the analytical method adopted here, any plant uptake of contaminants was observed. Generally speaking, the first explanation of the absence of plant uptake of organic contaminants in soil and sediments is the low bioavailability of the chemical structures. However, as also previously assessed, plant exudates might increase their bioavailability. In the frame of the results obtained here and considering that P. australis has been described as capable of absorbing chemical structures such as DDT, PCB [42], and nonylphenols [9], a role of the bacterial root endophytes in the recorded lack of quantization of PAHs in plant tissues cannot be excluded. Bacterial endophytes are reported as capable of significantly reducing evapotranspiration of toluene through aerial parts [43], as well as responsible for the degradation of naphthalene when the inoculated plants were grown in the naphthalene amended soil [44]. Results obtained here showed absence of PAH uptake by plants in presence of the onset of root endophytic metabolically active PAH degraders. The hypothesis merits a dedicated investigation in the near future that might open new perspectives in the correct management of plants exploited in phytoremediation because of the possibility of favoring the depletion of contaminants in the plant biomass produced during the process, eventually promoting safer composting processes. At the same time, the effect of* P. australis* as the sole factor eventually influencing the metabolic activity of the autochthonous microbial population for contaminant depletion, independently from the restoration of the resource-ratio framework [34], merits a dedicated investigation to eventually improve the cost sustainability of dredged sediment decontamination.

## 5. Conclusions

The effectiveness of the approach herein described requires to be verified at field scale; however, results obtained suggested that the biostimulation of autochthonous PAH degraders by the combination of N-spiking and phytoremediation can be a valuable approach for PAH depletion in contaminated dredged sediments. The vegetation of N spiked sediments with* P. australis* can be exploited as a sustainable approach for the acceleration of the process of PAH depletion and to favor the metabolism of GP PAH degraders. Since the metabolic activity of the GP degraders resulted in being mandatory for the depletion of the six condensed rings PAHs, the exploitation of* P. australis* resulted in being significantly beneficial. For the first time, a detailed description of the changes in the fractional number of metabolically active PAH degraders in rhizospheric and root endophytic bacterial populations has been reported. Results obtained suggested that, by using the sequence divergence of the PAH-RHD*α*, the biostimulation approach can be validated in relation to the nature of the PAH contamination. In the present validation effort, the biostimulation of the GP degraders resulted in being mandatory for the depletion of the high molecular weight PAHs and vegetation with* P. australis* resulted in being suitable to reach the goal. Moreover, it is worth interest that, with reference to the Italian law in environmental protection, the sediments at the end of the one-year treatment could be safely reallocated in commercial and/or industrialized area, at least for the admitted level of PAH contamination. As a net result, it is reasonable to propose the approach described here as exploitable at the dump site of sediment disposal and containment.

## Figures and Tables

**Table 1:** PAH concentration in sediment at the beginning of the experiment (BE) and after 12 months of incubation of N spiked sediments (Ns), 12 months of incubation of N spiked and vegetated sediments (NsV), and 12 months of incubation of not biostimulated sediment—control (notB). The mean values of the percentage of depletion (%) ±SD with respect to BE are reported on the base of the number of condensed rings and for the total PAHs (∑PAHs).

PAH	Abbr	BE (mg/Kg DW)	NsV (mg/Kg DW)	%	Ns (mg/Kg DW)	%	notB (mg/Kg DW)	%
2 condensed rings				79.97		45.49		ns
Naphthalene	NA	18.97 ± 4.72	3.80 ± 1.02		10.34 ± 3.11		18.89 ± 5.09	
3 condensed rings				68.14		34.44		
Acenaphthylene	ACE	14.67 ± 4.03	5.89 ± 1.08		9.87 ± 2.34		14.56 ± 4.34	
Acenaphthene	AC	33.87 ± 9.98	6.77 ± 2.12		21.89 ± 4.06		33.24 ± 9.19	
Fluorene	FL	19.78 ± 3.22	6.23 ± 0.96		12.52 ± 1.67		19.72 ± 3.03	
Phenanthrene	PH	19.23 ± 3.21	6.04 ± 0.99		11.90 ± 2.15		19.13 ± 4.09	
Anthracene	AN	14.98 ± 4.51	5.43 ± 0.94		10.59 ± 1.18		14.98 ± 4.12	
4 condensed rings				50.73		33.67		ns
Fluoranthene	FLU	15.57 ± 5.02	4.90 ± 0.91		9.03 ± 2.16		15.57 ± 5.10	
Pyrene	PY	12.87 ± 3.65	6.98 ± 1.09		8.01 ± 1.11		12.67 ± 3.06	
Benzo[a]anthracene	BaA	14.89 ± 4.65	9.03 ± 1.12		12.01 ± 1.07		14.76 ± 4.09	
Chrysene	CH	13.87 ± 3.24	7.03 ± 0.95		8.96 ± 0.91		13.61 ± 3.43	
5 condensed rings				36.65		23.57		ns
Benzo[b]fluoranthene	BbF	12.23 ± 3.12	7.89 ± 0.83		10.67 ± 1.14		12.09 ± 3.04	
Benzo[k]fluoranthene	BkF	7.89 ± 1.11	4.98 ± 0.82		4.98 ± 0.89		7.78 ± 0.94	
Benzo[a]pyrene	BaP	8.97 ± 1.14	5.43 ± 1.06		6.90 ± 1.07		8.94 ± 1.13	
Dibenz[a,h]anthracene	DA	7.98 ± 1.32	5.20 ± 1.12		6.20 ± 1.04		7.88 ± 1.16	
6 condensed rings				29.86		ns		ns
Indeno[1,2,3-cd]pyrene	PI	6.90 ± 2.21	4.89 ± 1.03	29.94	6.43 ± 2.05	ns	6.68 ± 2.12	
Benzo[g,h,i]perylene	BP	6.98 ± 2.03	4.90 ± 1.07	29.79	6.40 ± 2.07	ns	6.88 ± 2.09	

∑PAHs		229.67 ± 57.16	95.39 ± 17.11	58.47	156.66 ± 28.02	31.79	227.38 ± 56.02	ns

ns: not significant (*P* > 0.05).

**Table 2:** Bacterial groups and PAH-ring hydroxylating dioxygenases specific primers.

Target	Primers	Annealing T (°C)	PCR efficiency (%)	Reference
All groups	p1/p2	60	93	[30]
Gram positive	PAH-RHD*α* GP F/R	54	97	[6]
Gram negative	PAH-RHD*α* GN F/R	57	94	[6]
